# Reproducibility via coordinated standardization: a multi-center study in a *Shank*2 genetic rat model for Autism Spectrum Disorders

**DOI:** 10.1038/s41598-019-47981-0

**Published:** 2019-08-12

**Authors:** María Arroyo-Araujo, Radka Graf, Martine Maco, Elsbeth van Dam, Esther Schenker, Wilhelmus Drinkenburg, Bastijn Koopmans, Sietse F. de Boer, Michaela Cullum-Doyle, Lucas P. J. J. Noldus, Maarten Loos, Wil van Dommelen, Will Spooren, Barbara Biemans, Derek L. Buhl, Martien J. Kas

**Affiliations:** 1 Groningen Institute for Evolutionary Life Sciences, University of Groningen, Groningen, The Netherlands; 2 Neuroscience and Pain Research Unit, Pfizer Inc., Cambridge, MA USA; 3 Roche Innovation Center Basel, Basel, Switzerland; 4 Noldus Information Technology BV, Wageningen, The Netherlands; 5 Institut de Recherches Servier, Croissy-sur-Seine, France; 6 Janssen Research & Development, Janssen Pharmaceutica NV, Beerse, Belgium; 7 Sylics Synaptologics BV, Amsterdam, The Netherlands; 8 Department of Translational Neuroscience, University Medical Center Utrecht, Utrecht, The Netherlands

**Keywords:** Pharmacology, Autism spectrum disorders

## Abstract

Inconsistent findings between laboratories are hampering scientific progress and are of increasing public concern. Differences in laboratory environment is a known factor contributing to poor reproducibility of findings between research sites, and well-controlled multisite efforts are an important next step to identify the relevant factors needed to reduce variation in study outcome between laboratories. Through harmonization of apparatus, test protocol, and aligned and non-aligned environmental variables, the present study shows that behavioral pharmacological responses in *Shank2* knockout (KO) rats, a model of synaptic dysfunction relevant to autism spectrum disorders, were highly replicable across three research centers. All three sites reliably observed a hyperactive and repetitive behavioral phenotype in KO rats compared to their wild-type littermates as well as a dose-dependent phenotype attenuation following acute injections of a selective mGluR1 antagonist. These results show that reproducibility in preclinical studies can be obtained and emphasizes the need for high quality and rigorous methodologies in scientific research. Considering the observed external validity, the present study also suggests mGluR1 as potential target for the treatment of autism spectrum disorders.

## Introduction

The alarmingly high estimate of failure (50–80%) to replicate findings in preclinical studies is a prevalent issue of great scientific and public concern that needs to be addressed^1–3^. While the lack of reproducibility of scientific findings has gained significant attention, thus far not many attempts and strategies have been implemented to tackle this challenging situation. Given that the ability to replicate empirical findings is a prerequisite of experimental science, deficient reproducibility hinders scientific credibility and progress. For biomedical animal research in particular, poor reproducibility questions the benefit of research in the ethical analysis of animal experiments^4^, prevents pharmacotherapeutic development, and results in great monetary loss^5,6^. The inability to replicate scientific findings points toward systematic inefficiencies in the way studies are planned, executed, analyzed, and reported.

Although drivers of data variability across different research sites are not well understood, the use of different animal strains, housing, husbandry, and testing environments, and/or different lab standard operating procedures (SOPs) are generally considered critical factors^7,8^. Therefore, rigorous genetic (animals) and environmental (housing, husbandry, testing procedures) standardization have been advocated as good laboratory practice to reduce variation in experimental results^9^. However, excessive standardization results in more homogeneous study populations, which in turn generates spurious results as they are representative to the specific standardized conditions under which the data was obtained, thereby hampering replicability^8,10–12^.

Gene-environment interactions can considerably affect animal behavior. As laboratories differ in many factors (personnel, odors, noise, microbiota, etc.) and the intrinsic variability of the animals assessed is high (i.e. genetic variation from different vendors), the variation of phenotypes between laboratories, even in the same genetic strain of animals, is generally much larger than the variation within laboratories as clearly shown by the landmark multi-laboratory study of Crabbe *et al*.^13^. Then, contrary to common belief, excessive standardization, understood as controlled environmental enrichment that decreases biologically meaningful variation, doesn’t contribute to highly reproducible results.

To improve reproducibility of preclinical studies and maximize the chances of discovering meaningful treatment effects or fundamental biological principles, several suggestions have been proposed^6,14,15^; in particular, to take into account unavoidable between-laboratory variations. For this, the use of multi-laboratory study designs has been advocated as a valuable approach to evaluate the influence of heterogenization between different laboratory settings on data variability^8^. Using the genetically modified *Shank2* knockout (KO) rat model of synaptic dysfunction relevant to autism spectrum disorders reported to exhibit autistic-like hyperactive and repetitive behavioral phenotype^16^, the primary objective of the current study was to investigate whether these previously reported results could be reproduced and replicated across three study sites by following the same experimental protocol for behavioral evaluation with automated video scoring analysis and drug testing. To reduce the impact of environmental factors that typically differ greatly between laboratories and are difficult to control, an identical test setup, i.e. a PhenoTyper® cage and EthoVision XT 12 video tracking software (Noldus Information Technology BV; Wageningen, The Netherlands) was used at all sites. *Shank2* KO rats were placed in this novel environment in an attempt to reproduce the previously observed hyperactive and repetitive behavioral phenotype of these animals^16^ that recapitulate the characteristic behavioral abnormalities of autism spectrum disorder (ASD) in humans. In addition, to confirm the normalization after pharmacological treatment with the metabotropic Glutamate Receptor 1 (mGluR1) antagonist JNJ16259685, we included a dose-response of the drug treatment to strengthen interpretation for the effect. Finally, as a secondary objective, a comparison between 2 behavioral scoring methods to evaluate the phenotype (i.e., automated versus manual scoring) was performed using the same recorded videos.

## Materials and Methods

### General study design

Based on the demonstration of hyperactivity and repetitive circling behaviors observed in the *Shank2* KO rat model^16^, a cross-site study focusing on these behaviors was initiated. Specifically, we aimed to assess the behavioral phenotype, as well as the pharmacological effects in both *Shank*2 KO and littermate controls (WT) using automated video scoring. A phenotypic assessment was carried out quasi-simultaneously (i.e. during the same month) in three different research facilities. Although the aim of this study was to explore the reproducibility of the results, it was not intended to fully reproduce the original methodology; standardized phenotyping equipment was used, and small changes were made to the protocol for this study.

To optimize the chance of successful replication, the protocol entailed controlling several aspects of the study design from animal provider and shipment, to details of experimental procedures. In addition, some other factors were not harmonized across sites presumably revealing the robustness of the study results.

To enable consistency in the environmental aspects of the behavioral assays and automated scoring methods between sites, and in addition to the PhenoTyper chambers provided, the operational definition of the behavioral categories to score were aligned across sites to pursue consistency in the manual scoring (e.g. what represents a turn). Janssen Pharmaceutica NV (Beerse, Belgium) provided the mGluR1 antagonist JNJ16259685^15,17^ to all sites to eliminate variability in pharmacological outcomes due to inconsistencies of the chemical batch (e.g. differences in purity).

### Laboratories

The experiment was conducted at the Groningen Institute for Evolutionary Life Sciences (GELIFES) of the University of Groningen (RUG, Groningen, The Netherlands), the Neuroscience and Pain Research Unit of Pfizer Inc. (Cambridge, MA, USA), and at the Roche Innovation Center Basel (Basel, Switzerland).

### Study design

The experiment was carried out during four consecutive weeks of four testing days per week (Monday to Thursday), always starting 4 hours after onset of light.

A full crossover design was followed, so that each subject received each dose once with a one-week wash-out period before the next dosing and testing; for this, on each day of the week 3 WT and 3 KO were tested, so that at the end of the week all subjects (12WT/12KO) were tested once with one of the four treatments (including vehicle).

On a given testing day, each subject was weighed and given a single-dose injection of either vehicle (saline) or JNJ16259685 (0.02, 0.04, or 0.63 mg/kg in 5 ml/kg volume), administered subcutaneously in the flank. The dosing order was alternated between genotypes and counterbalanced across days. The treatment conditions were randomized throughout the experiment with a Latin-square design.

Thirty minutes after dosing, the subject was placed in a PhenoTyper chamber and video-recorded for 30 minutes after which the chamber was cleaned with alcohol wipes. The behavior was scored after the experiment from the video images using EthoVision XT 12 for the automated scoring or The Observer XT 13 for the manual scoring. For the latter, the observer was blinded to genotype and treatment.

### Animals

36 Sprague-Dawley male rats carrying a targeted deletion of the *Shank2* gene (KO) and 36 male WT rats matched for age were generated as described by Modi *et al*., 2018. A batch of 12 KO and 12 WT rats was shipped from Charles River Laboratories (Wilmington, MA, USA) to each of the three sites involved. Animals had at least ~4 weeks of habituation to their housing facility and were around 3 months old at the start of the experiment.

Animals had *ad libitum* access to food and water with a similar 12:12 light-dark cycle at all three sites.

All animal procedures were carried out following the regulations of the Directive 2010/63/EU and in accordance with the recommendations of the Guide for the Care and Use of Laboratory Animals. The protocol was approved by the Pfizer Institutional Animal Care and Use Committee, the Basel Cantonal Animal Protection Committee adhering to Swiss federal legislation, and the University of Groningen’s Animal Welfare Body in accordance with the Central Committee for Animal Experiments.

### Drug

The test compound, JNJ16259685-AAA (3,4-dihydro-2H-pyrano[2,3]b-quinolin-7-yl) (cis-4-methoxycyclohexyl) methanone, is a brain penetrant selective mGluR1 antagonist with an affinity of 0.34 nM (Ki value) for rat mGlu1 receptor, which potently and completely inhibits glutamate-induced increases in intracellular Ca2+ concentrations with an IC50 value of 3.24 nM (Lavreysen *et al*. 2004). The compound was synthesized at Janssen Pharmaceutica NV and centrally shipped to the participating labs, by the Compound Logistics & Formulation unit at Janssen Pharmaceutica NV. All sites used compound from the same chemical batch.

JNJ16259685-AAA was dissolved in saline (i.e. H2O + HCL + NaCL to reach a pH of 7.4) and serial dilutions were made for the different doses.

### Equipment and software

A PhenoTyper 4500 behavioral assessment chamber (Noldus Information Technology BV) was shipped to each of the three sites, to ascertain standardization of behavioral recording and analysis. The PhenoTyper 4500 chamber includes a black square arena (floor area 45 × 45 cm), 4 matted walls with ventilation holes at the top (66 cm tall). The top unit serves as a lid from which only the infrared sensitive camera (30 fps at 640 × 480 resolution using NTSC format) and the 3 arrays of dimmable infrared LED lights were used.

Automated scoring of rat’s behavior was done using EthoVision XT 12 video tracking software, including the Rat Behavior Recognition module (Noldus Information Technologies BV) which allowed a repeatable, objective, and consistent analysis of the 30 minutes video. Details of the acquisition settings are listed in Table 1 of the Supplementary material.

**Table 1:** Description of the detection criteria for the automated tracking using EthoVision XT 12.

Variable	Description
Circling	Rotation based on direction from tail-base to center-point, clockwise, count every 0.75 rotation, threshold 30 degrees (frequency)
Circling 2	Rotation based on direction from tail-base to center-point, counterclockwise, count every 0.75 rotation, threshold 30 degrees (frequency)
Rearing Supported	Probability greater than 50%, excludes instances shorter than 0.50 s (frequency, cumulative duration)
Rearing Unsupported	Probability greater than 80%, excludes instances shorter than 0.50 s (frequency, cumulative duration)
Movement	Averaging interval of 1 sample, start velocity 5.00 cm/s, stop velocity 1.00 cm/s based on the body center-point (mean, cumulative duration)
Walking	Probability greater than 10%, exclude instances shorter than 0.00 s (frequency, cumulative duration)

The video files that were run offline through EthoVision XT 12, were scored using The Observer XT 13 software by a blinded scorer at each of the three sites; for this, only the second 10-minute bin was analyzed.

### Behavioral readouts (automated and manual)

Following a predetermined set of criteria (Tables 1 and 2), behavior was analyzed using two methods of scoring. The predetermined criteria were discussed and agreed upon in detail by the three sites.

**Table 2:** Description of the behavioral definitions used for the manual scoring with The Observer XT 13.

Behavior	How to manually score rat behavior in The Observer XT
Circling (total time)	Start scoring when the rat moves in rapid circles in the same direction lacking apparent goal or function, do not stop until the rat finishes circling. Don’t score each full rotation separately, score it as a bout
Circling (frequency)	Score each event when the rat turns in a full circular motion (as reference, the nose has to travel at least 270 degrees)
Rearing Unsupported/Supported	Start to score this behavior when the rat puts its weight on its hind legs, raises its forepaws from the ground, and extents its head upward. Its forepaws can either lean on the wall or stay suspended
Inactive	The rat is sitting still on the floor, without performing any of the other scored behaviors, and showing from little to no movement based on the rat’s body center-point
Walking	Start behavior when the rat’s body center-point begins to move

#### Automated scoring

Table 1 lists the behaviors and their definition as recognized by EthoVision XT 12. The automated scores of these behaviors are based on the entire 30 minutes of the experimental session.

#### Manual scoring

The manual scoring of behaviors was done by a trained observer blind to genotype and treatment, using The Observer XT 13. Table 2 depicts the behavioral definitions on which the scoring was based. This scoring was only carried out for the second 10-minute bin of the experimental session (i.e. from minute 11 to minute 20).

### Data management

The data were archived on the cloud platform of Sylics (Synaptologics; Amsterdam, The Netherlands) which allowed us to share video files, raw data, and spreadsheets between the three sites in an efficient and secure way.

### Statistics

The behavioral outcomes were analyzed using SPSS according to the different objectives defined:

#### Reproducibility across sites

A three-way ANOVA with genotype (two levels) and site (three levels) as between-subject factors, and treatment as the repeated within-subject factor [four levels (vehicle, 3 doses)] was performed on absolute data values for each of the readouts. In the case of a main site and genotype effect in this absolute data set, normalized values (relative to vehicle treatment) were analyzed using the same three-way ANOVA design. This analysis aimed to address reproducibility across sites in terms of the phenotype evaluation as well as the pharmacological intervention.

#### Method of scoring

To explore the effect of different methods of scoring, manual scored data was compared to automated data using the same three behavioral outcomes (walking, rearing and circling). Only the middle 10-minute bin of the entire 30-minute observation period was analyzed by employing a 4-way ANOVA with genotype and site as between-subject factors, and method of scoring [two levels: manual (The Observer XT 13) and automatic (EthoVision XT 12)] and treatment as repeated within-subject factors.

## Results

### Standardization across sites

To ensure high-quality data, the protocol shared across sites addressed randomization and blinding principles in addition to detailed environmental variables, handling and testing procedures summarized in Table 3. The experimental protocol for this study was based on a previous single-site study using the same compound and different automated scoring equipment^16,17^. The study design was then discussed between the consortium partners to address alignment of factors with anticipated higher relevance to maximize the power of the study. A summary of aligned and non-aligned factors is presented in Table 3.

**Table 3:** Summary of the experimental factors that were aligned across sites.

Factor	RUG	Pfizer	Roche	Aligned?
Provider of animals	Charles River	Charles River	Charles River	Y
Age at start of experiment	~3 months	~3 months	~3 months	Y
Average bodyweight at the start of experiment	WT 407gr (±33)/KO 399gr (±45)	300–350gr	WT 428 gr (±32)/KO 377gr (±33)	N
**Animal-related guidelines**				
Housing	Single housed	Single housed	Single housed	Y
Cage size	Makrolon type 2 L	Innovive Rat Cage	Makrolon type IV	N
Bedding	Lignocel BK8/15	Alpha Dri	Lignocel FS-14	N
Food type	Standard Altronim rodent chow	Standard Purina rat chow (5053)	KLIBA NAFAG 3436	N
Cage cleaning	1/week Friday	1/week Friday	1/week Friday	Y
Enrichment	Wooden bar, nesting material (Enviro-dry)	Plastic bone, nesting material (Bed-R’Nest)	Wooden bar, nesting material	Y^#^
Handling	Tail	tail	Body	N
Experimenter	Master student (MAA)	Undergraduate Researcher (MCD)	Laboratory Associate (MM)	N
Gloves	Yes	Yes	Yes	Y
Disturbance	other rats housed	Not applicable	Radio (60 dB)	N
Identification	Cage card, ear-clip and tail mark	Cage card and tail mark	Cage card	N
**Physical environment of the housing room (HR)**				
Humidity	42%	45%	50%	N
Temperature	73πF	72πF	70πF	N
Lighting	~35 Lux	~35 Lux	~150 Lux	N
**Behavioral testing**				
Testing days	Mon-Thur.	Mon-Thur.	Mon-Thur.	Y
Test room	Separate	Same as Holding room (HR)	Same as HR	N
Lighting in procedure room	~35 lux	~35 Lux	~150 Lux	N
Volume	35–40 dB	60 dB	60 dB	N
Temperature	73 °F	72 °F	70 °F	N
Humidity	42%	45%	50%	N
Randomization	Latin-square	Latin-square	Latin-square	Y
Sample size per genotype	11 WT/12 KO	12 WT/12 KO	12 WT/12 KO	Y*
Dosing	s.c. in the flank	s.c. in the flank	s.c. in the flank	Y
	in holding room	in procedure room	in procedure room	N
Post-dosing time	30 min	30 min	30 min	Y
**Environment and Equipment**				
PC	outside procedure room	in procedure room	in procedure room	N
Experimenter present	No	yes, behind blinders	Yes	N
Equipment	PhenoTyper 4500	PhenoTyper 4500	PhenoTyper 4500	Y
Light inside PhenoTyper	~14.5 Lux	~14 Lux	~80 Lux	N**
Cleaning	alcohol wipes	alcohol wipes	alcohol wipes	Y
**Software**				
Automated scoring	EthoVision XT 12	EthoVision XT 12	EthoVision XT 12	Y
Manual scoring	The Observer XT 13	The Observer XT 13	The Observer XT 13	Y
Blinded scoring	yes	yes	Yes	Y
**Compound**				
Provider	Janssen Pharmaceutica NV	Janssen Pharmaceutica NV	Janssen Pharmaceutica NV	Y

*One rat missed the last dosing so it was excluded from the analyses. ^#^Home cage enrichment was agreed to be applied at all three sites but adhered to institutional standard practices. **Light intensity inside the chamber was aligned but technical aspects prevented one of the sites to use the agreed intensity.

### Behavioral evaluation

The behavioral read-outs were (1) circling behavior, expressed as the frequency of circling (clockwise and counterclockwise), (2) rearing, expressed as frequency of supported and unsupported rearing, and (3) time spent walking. These behavioral read-outs were analyzed in separate ANOVA’s.

#### Hyperactive and repetitive phenotypes of *Shank*2 KOs were consistently observed across study sites

**Figure 1:**
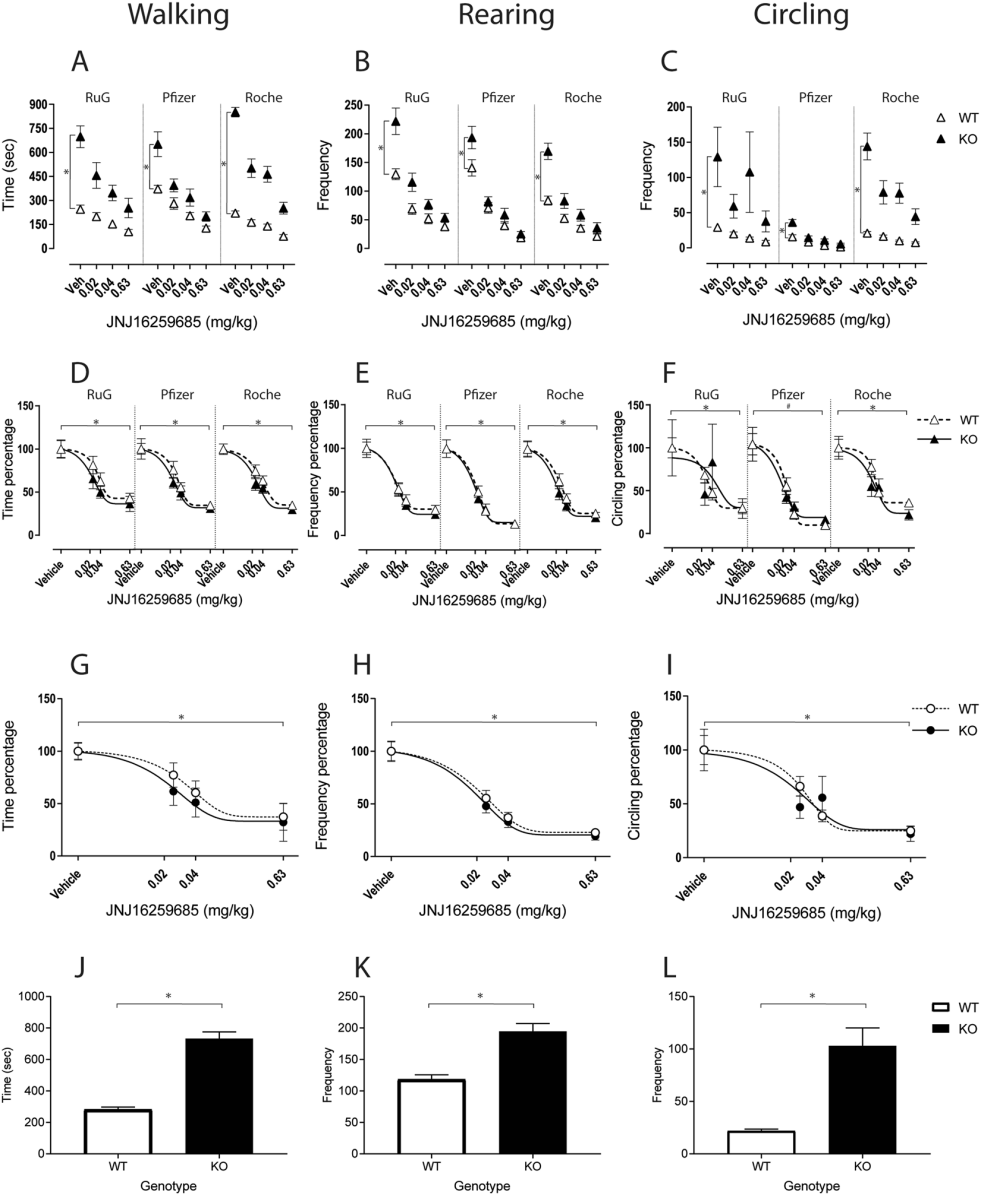
(**A**–**C**)Absolute values for walking (**A**), rearing (**B**), and circling (**C**) across the three sites from automated scoring analysis (full 30’ session). (**D**–**F**) Normalized values relative to the vehicle for walking (**D**), rearing (**E**), and circling (**F**) across the three sites. The averaged site values for the respective dose levels (depicted in **D**–**F**) are shown in (**G**–**I**). Pooled data from the three sites under vehicle condition for both genotypes for walking (**J**), rearing (**K**), and circling (**L**) behavior. All values are expressed as mean ± S.E.M. *Statistically significant differences (*p < 0.05*), ^#^Points out trend (*p = 0.06*).

Analysis of the automated scorings (EthoVision XT 12), during the 30-minute PhenoTyper chamber exposure, revealed that *Shank*2 KO rats showed increased walking (Fig. 1A; *genotype F(1,65)* = *94.95, p* < *0.001*), rearing (Fig. 1B; *genotype F(1,65)* = *35.9, p* < *0.001)*, and circling behavior (Fig. 1C; *genotype F(1,65)* = *22.69, p* < *0.001*) relative to the WTs across all three study sites. However, a significant genotype x site interaction, and genotype x treatment interaction effect was observed for walking (*F(2,65)* = *5.9, p* < *0.005;* (*F(3,195*) = *29.9, p* < *0.001)* and circling (*F(2,65)* = *3.0, p* < *0.05; F(3,195)* = *5.6, p* < *0.001*) while for rearing only the latter interaction reached significance (F(3,195) = 13.5,p < 0.001). In addition, a significant overall site effect for rearing (*F(2,65)* = *7.1, p* < *0.005*) and circling *(F(2,65)* = *3.6, p* < *0.05*) was found. Univariate ANOVA of only the vehicle data showed a significant genotype effect for walking, rearing and circling across all three sites (Fig. 1A–C). To display the hyperactive and repetitive behavioral phenotype of *Shank*2 KO rats, the vehicle data from all three sites were pooled for both phenotypes (Fig. 1J–L) and analyzed with ANOVA (walking: F(1,70) = 48.9, *p < 0.001*; rearing: F(1,69) = 31.8, *p < 0.001*; circling: F(1,70) = 55.6, *p < 0.001*).

Further analyses suggested that the site x genotype interaction effect for walking was explained by the higher Pfizer scores of the WT group compared to the RUG and Roche values (*F(2,32)* = *13.98, p* < *0.001*). The site effect for rearing was due to the lower Roche scoring values of both groups of animals compared to the RUG and Pfizer data sets *(F(2,65)* = *7.12, p* < *0.005*). The site and site x genotype interaction effect for circling was mainly caused by the general lower Pfizer values of, primarily, the *Shank*2 KO group (*F(2,33)* = *5.19, p* < *0.02*) and mildly by the WT (*F(2,32)* = *22.58, p* < *0.001*) compared to the RUG and Roche values. Yet, all these study-site and genotype main- and interaction-effects disappeared when normalizing the raw data by expressing them as relative to the vehicle control (Fig. 1D–F, Supplementary Table 2).

#### Consistent dose-dependent attenuation of motor activity and circling behavior in *Shank2* KO and WT rats by JNJ16259685 treatment across study sites

JNJ16259685 treatment resulted in a robust dose-dependent suppression of walking, rearing, and circling behavior in both WT and *Shank*2 KO rats at all three study sites. For all three behavioral parameters, a significant overall main effect of treatment was found (walking (Fig. 1A): *F(3,195)* = *125.3, p* < *0.001)*, rearing (Fig. 1B): *F(3,195)* = *192.6, p* < *0.001)*, and circling (Fig. 1C): *F(3,195)* = *12.19, p* < *0.001*) as well as a significant treatment x genotype interaction (Supplementary Table 2). This interaction effect is predominantly caused by the robustly enhanced hyperactivity and repetitive circling behavior of the KO animals and completely disappears when normalizing the raw data by expressing them as relative to the vehicle condition (Fig. 1D–F, Supplementary Table 2). This indicates that the JNJ16259685 treatment effects were similar for KO and WT rats and, importantly, consistent across all three sites. Combining the data from all three sites (Fig. 1G–I) demonstrated similar dose-response curves for JNJ16259685 treatment to inhibit walking (ID_50_ = 0.9549 and 0.583 for WTs and KOs, respectively), rearing (ID_50_ = 0.6755 and 0.6883 for WTs and KOs respectively), and circling behavior (ID_50_ = 1.034 and 0.535 for WTs and KOs respectively) for both WT and *Shank*2 KO animals.

#### Treatment effects are comparable whether scored automatically or manually

**Figure 2:**
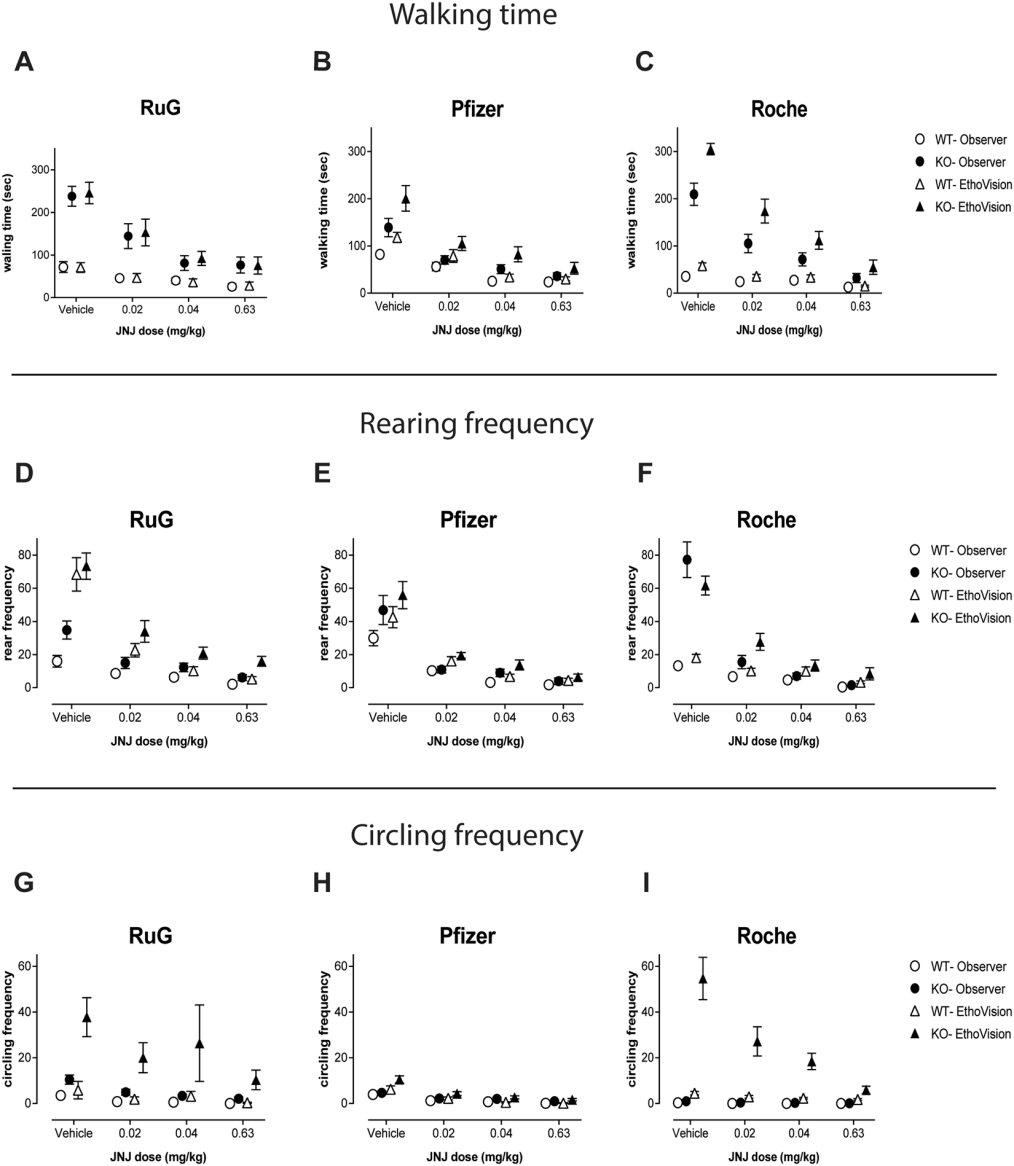
Absolute values across treatments for walking time (**A**–**C**), rearing frequency (**D**–**F**), and circling frequency (**G**–**I**) for the second 10-minute bin of the automated (triangles) and manual scored (circles) data, comparing the KO (black shapes) and WT (white shapes) rats. All data points express the mean ± S.E.M.

The present study was focusing on the reproducibility of automated scored behavior from a previous study^16^. To test whether the level of reproducibility of the automated scoring was comparable to that of manual scoring, a method frequently used in behavioral pharmacology studies, we compared manual and automated scored behaviors in a 10-minute segment of the data across sites. Manual (The Observer XT 13) and automated (EthoVision XT 12) scorings of the second 10-minute bin of the recordings were employed and included in the ANOVA as an additional (within-subject) factor; this 10-minute time segment was selected because it had the highest rate of activity in the 60-minute evaluation of Modi *et al*., 2018. A four-way ANOVA analysis revealed a significant main effect of method for all three behavioral parameters (see Fig. 2A–C for walking; Fig. 2D–F for rearing, and Fig. 2G–I for circling, and Supplementary Table 3) as well as several method interaction effects with genotype, site, and treatment (see Supplementary Table 3).

Overall, the automated EthoVision-scored values for walking, rearing, and circling were higher than the manual Observer-scored values (*F(1,65)* = *112.1*, *p < 0.001*; *F(1,65)* = *47.8, p* < *0.001; and F(1,65)* = *52.9, p* < *0.001*; respectively). The hyperactive and repetitive circling behavioral phenotype of the KOs as well as its dose-dependent suppression by JNJ16259685 treatment were reliably detected at all three study sites by manual scoring (Walking: Fig. 2A–C; Rearing: Fig. 2D–F; Circling: Fig. 2G–I). Interestingly, while manual- and automated-scored circling displayed visibly different scores across sites, no significant main or interaction effects appeared following normalization of the raw data relative to the vehicle condition. However, for the normalized rearing data for the 10-minute bin (data not shown), a significant main effect of method and method x treatment interaction persisted (Supplementary Table 3). Further analyses revealed higher values at the JNJ16259685 0.04 and 0.63 mg/kg treatments for the automated method relative to the manual scoring method.

## Discussion

The present study demonstrates that rigorous alignment of experimental protocols between three research centers resulted in comparable experimental findings across sites for both genotype and treatment effects. The phenotypic difference between the *Shank2* KO and WT rats was reliably observed in all three study sites. While there were differences in amplitude of the genotype effect on behavior, all three sites observed that the KO rats displayed consistently heightened motor activity (i.e. walking and rearing) and stereotypic circling behavior when compared to WT rats. This finding indicates reproducible findings across sites, in addition to the replication of the original report^16^. Importantly, our data demonstrates the robustness of the *Shank2* deletion-induced behavioral phenotypes that may mimic some of the behavioral abnormalities observed in ASD. Likewise, a consistent and dose-dependent attenuation of motor activity and circling behavior in both KO and WT rats by JNJ16259685 was found across the three study sites.

This high level of reproducibility is likely to be attributed to the rigorously standardized experimental protocol. The study design employed herein was adapted from the original work of Modi *et al*., 2018 with particular effort to prevent bias in the design, collection, and analysis of data (e.g. blinding, randomization, carry-over effects, etc.); and to analyze the data similarly through automated scoring. Besides the study design, experimental conditions that may have biological relevance to the expression of the phenotype (e.g., age of the animals) were aligned across sites. Conversely, factors that were not expected to have direct biological relevance related to phenotype expression were addressed; however, at a variable level between sites (e.g., environmental enrichment applied for all sites, but the level of environmental enrichment for the housing conditions differed across sites). Thus, environmental variability between the three sites was allowed, which introduced heterogenization for experimental conditions that were site specific^10,18^. Therefore, our study appears to support the assumption that a combination of standardized and heterogenized factors can lead to a high level of reproducibility between different laboratories. Selection of these factors may be dependent on study aim and neurobiological construct that is being investigated; indeed, for future study designs, it is recommended to carefully review the standardization of environmental factors and consider their relevance in light of the phenotype of interest. For example, by over-standardizing only factors that are not biologically relevant to the expression of the (behavioral) phenotype of interest, the result is at risk of being highly idiosyncratic. On the other hand, and as recently suggested, introducing systematic heterogenization of certain factors can boost external validity and thus reproducibility^19^.

Preclinical studies are a stepping stone in the pipeline for new pharmacotherapeutic treatments of human disorders. Thus, the development and assessment of animal models that recapitulate specific phenotypes of the disorders in a consistent manner is crucial when testing new therapeutic targets. In addition to protocol alignment for factors related to the laboratory (micro-)environment, selection of the type of animal model is also important in view of reproducibility (e.g., when the originally observed effect sizes in outcome measures for the selected model are small).

Here, the initial hyperactive and repetitive behavioral phenotypes of *Shank2* KO rats were robust as these behavioral alterations are consistently observed across various different *Shank*-mutations in both rats and mice under a variety of experimental testing conditions^20–25^ suggesting the authentic relevance of these postsynaptic scaffolding proteins that are present at glutaminergic synapses for ASD-like behaviors and the suitability for pharmacological testing. Nonetheless, attention must be drawn to the different underlying circuitry responsible for the robust phenotype since it might not completely overlap across the different *Shank* mutations, as previously reported by Yoo *et al*. 2014 who found inconsistencies in molecular, physiological and behavioral data between and within the *Shank* mutant mouse lines^26^.

Recapitulating and expanding on the findings from Modi *et al*. (2018), administration of the selective and high-affinity mGluR1 antagonist JNJ16259685 effectively attenuated the hyperactivity and repetitive circling behavior of *Shank*2 KO rats in a dose-dependent manner. While Modi *et al*. demonstrated a significant attenuation of these behavioral phenotypes in both WT and KO animals, they argued that JNJ16249685 (0.63 mg/kg) normalized KO behavior to WT vehicle-dosed levels. Here, we show that the locomotor-suppressing effects of JNJ16259685 produce similar dose-effect curves in both genotypes. This goes well in line with the fact that the mGluR1 receptors are richly distributed in regions associated with motor function including the cerebellum^27,28^ and basal ganglia^29^, and are believed to play an important role in movement, motor coordination, and motivation^30,31^. Our findings agree with the results of Hodgson *et al*., 2011, who reported a dose-dependent reduction in novelty-induced locomotor and rearing activity of Wistar rats. Hence, they support the assertion that the mGluR1 is involved in general motivation to explore their environment^31^. Although this study was focusing on reproducing the behavioural features in the Shank2 KO rats, electrophysiological characterization can be reviewed in Modi, *et al*. (2018). Overall, our results support the suggestion that the hyperactive phenotype of *Shank2*-deficient rats is associated with enhanced striatal mGluR1 signaling^16^ thereby providing face, construct and predictive validity of this animal model for ASD. This study also suggests mGluR1 as potential target for the treatment of ASD.

Another aim of this study was to compare two different methods of scoring behavior, automated versus manual scoring. Behavioral studies are relevant for most biological, evolutionary, and biomedical research questions, creating a need for high-throughput experiments and mechanistic insight; however, the effort and time spent in manual scoring and data processing becomes a burden when conducting behavioral experiments. Therefore, there is a need for an automated screening of an animal’s behavior capable to discriminate between different behavioral categories, especially in the presence of animal manipulations. For the current study, three behavioral categories were chosen to compare between an automated and a manual scoring; these categories have a different level of complexity in terms of how straightforward it is to score the behavior. The selected categories are walking time, rearing frequency and circling frequency, from the most to least simple.

Overall, the automated scoring showed higher rates compared to the manual scoring at all three sites. The mismatch between methods was present for both genotypes and across treatments indicating that the differences between methods might originate from the flexibility of the behavioral definition adopted for each scoring method; in addition, discrepancies might also be attributed to the human observer ‘smoothing’ the scoring, meaning that brief intervals between behaviors are scored as the continuity of the behavior, while the automated scoring counts separate events. This suggests that special attention must be drawn not only to the definition of the behavior being scored, but also to the parameters that frame this definition, likely in this case smoothing by the human scorer and the continuity of the behavior scored as separate events by the automated scoring. These parameters have to be adapted according to the behavior being defined and the instrument used. Moreover, the concordance between methods was higher for the simplest behavioral category (walking time) and the lowest for the most complex category (circling frequency) suggesting that the coherence between scoring methods is more easily attainable when the behavioral category is unambiguous. Importantly, both the manual and automated scoring methods succeeded in detecting the phenotype and treatment effects (Supplementary Table 3), suggesting that they are both reliable methods to assess the relatively simple behaviors scored in the current study.

To conclude, by using a combination of standardization and heterogenization for experimental factors, a harmonized protocol was generated and applied to a multicenter study in which genotype and treatment effects were studied at a behavioral level. Here we showed that, following careful alignment of these factors, reproducibility of genotype and treatment effects in rodents can be established for both automated- and manually-scored behaviors.

## Supplementary information

Supplementary tables
