# Supplementary material for: Reproducibility via coordinated standardization: a multi-center study in a Shank2 genetic rat model for Autism Spectrum Disorders
Source: Sci Rep. 2019 Aug 12;9:11602. doi: 10.1038/s41598-019-47981-0 (PMC6690904; doi:10.1038/s41598-019-47981-0)
Supplement: Supplementary file 1 — Supplementary tables [file 41598_2019_47981_MOESM1_ESM.pdf]

Reproducibility via coordinated standardization: a multi-center study in a S*hank*2 genetic rat model for Autism Spectrum Disorders

María Arroyo-Araujo, Radka Graf, Martine Maco, Elsbeth van Dam, Esther Schenker, Wilhelmus Drinkenburg, Bastijn Koopmans, Sietse F. de Boer, Michaela Cullum-Doyle, Lucas P.J.J. Noldus, Maarten Loos, Wil van Dommelen, Will Spooren, Barbara Biemans, Derek L. Buhl, and Martien J. Kas

Supplementary Table 1

Detection settings for video tracking and automated scoring of the video recordings in EthoVsion XT 12. * The value differed within this range across-sites according to the particular needs for proper detection of the animal.

**Method**
Dynamic Subtraction
Simple Model/Rodents
Subject color: Brighter
Bright: 70-225
Frame weight: 1
**Smoothing**
Video pixel smoothing: low
Track noise reduction: off
**Subject Contour**
Erosion: 2
Dilation: 2 or 3*
**Subject Size**
Minimum: 1000-3000*
Maximum: 12000-14000*

Supplementary Table 2

Three-way ANOVA results of the automated 30 min behavioral scores.

	**Walking**	**Walking normalized**	**Rearing**	**Rearing normalized**	**Circling**	**Circling normalized**
**Treatment**	F (3,195) =125.3 P<0.001	F (3,195) =118.8 P<0.001	F (3,195) =192.6 P<0.001	F (3,195) =205.2 P<0.001	F (3,195) =12.19 P<0.001	F (3,195) =43.91 P<0.001
**Genotype**	F (1,65) =94.9 P<0.001	F (1,65) =2.96 NS	F (1,65) =35.9 P<0.001	F (1,65) =1.05 NS	F (1,65) =22.69 P<0.001	F (1,65) =0.023 NS
**Site**	F (2,65) =0.34 NS	F (2,65) =0.3 NS	F (2,65) =7.1 P<0.005	F (2,65) =0.17 NS	F (2,65) =4.25 P<0.02	F (2,65) =1.62 NS
**Treatm x Geno**	F (3,195) =29.9 P<0.001	F (3,195) =1.7 NS	F (3,195) =13.5 P<0.001	F (3,195) =29.9 NS	F (3,195) =5.57 P<0.01	F (3,195) =2.36 NS
**Treatm x Site**	F (6,195) =0.81 NS	F (6,195) =0.43 NS	F (6,195) =2.5 P<0.05	F (6,195) =0.59 NS	F (6,195) =1.28 NS	F (6,195) =1.17 NS
**Geno x Site**	F (2,65) =5.9 P<0.005	F (2,65) =0.03 NS	F (2,65) =1.32 NS	F (2,65) =0.03 NS	F (2,65) =3.6 P<0.05	F (2,65) =0.15 NS
**Treatm x Geno x Site**	F (6,195) =1.48 NS	F (6,195) =0.03 NS	F (6,195) =0.48 NS	F (6,195) =0.23 NS	F (6,195) =0.15 NS	F (6,195) =0.34 NS

Supplementary Table 3

Four-way ANOVA results of the 10-minute bin manual (The Observer XT 13) and automated (EthoVision XT 12) behavioral scores.

	**Walking**	**Walking normalized**	**Rearing**	**Rearing normalized**	**Circling**	**Circling normalized**
**Treatment**	F (3,195) =108.9 P<0.001	F (3,195) =86.9 P<0.001	F (3,195) =157.4 P<0.001	F (3,195) =154.6 P<0.001	F (3,195) =20.4 P<0.001	F (3,195) =22.9 P<0.001
**Genotype**	F (1,65) =85.69 P<0.001	F (1,65) =2.69 NS	F (1,65) =63.2 P<0.001	F (1,65) =1.8 NS	F (1,65) =23.5 P<0.001	F (1,65) =0.75 NS
**Site**	F (2,65) =1.52 NS	F (2,65) =0.45 NS	F (2,65) =2.7 NS	F (2,65) =0.95 NS	F (2,65) =6.4 P<0.005	F (2,65) =0.09 NS
**Method**	F (1,65) =112.1 P<0.001	F (1,65) =0.66 NS	F (1,65) =47.8 P<0.001	F (1,65) =16.4 P<0.001	F (1,65) =52.9 P<0.001	F (1,65) =2.7 NS
**Treatm x Geno**	F (3,195) =32.5 P<0.001	F (3,195) =0.76 NS	F (3,195) =31.7 P<0.001	F (3,195) =1.2 NS	F (3,195) =6.7 P<0.01	F (3,195) =0.33 NS
**Treatm x Site**	F (6,195) =0.57 NS	F (6,195) =0.81 NS	F (6,195) =-.08 NS	F (6,195) =0.48 NS	F (6,195) =1.28 NS	F (6,195) =0.13 NS
**Treatm x Site x Geno**	F (6,195) =3.98 P<0.002	F (6,195) =0.93 NS	F (6,195) =3.9 P<0.002	F (6,195) =0.69 NS	F (6,195) =2.49 P<0.05	F (6,195) =0.15 NS
**Method x Site**	F (2,65) =21.12 P<0.001	F (2,65) =1.09 NS	F (2,65) =3.4 P<0.05	F (2,65) =2.39 NS	F (2,65) =9.54 P<0.001	F (2,65) =1.45 NS
**Method x Geno**	F (1,65) =34.54 P<0.001	F (1,65) =6.4 P<0.02	F (1,65) =0.04 NS	F (1,65) =3.09 NS	F (1,65) =28.49 P<0.001	F (1,65) =1.1 NS
**Method x Site x Geno**	F (2,65) =8.35 P<0.002	F (2,65) =2.65 NS	F (2,65) =0.15 NS	F (2,65) =1.36 NS	F (2,65) =6.23 P<0.002	F (2,65) =0.64 NS

**Geno x Site**	F (2,65) =6.26 P<0.001	F (2,65) =0.59 NS	F (2,65) =4.9 P<0.02	F (2,65) =0.8 NS	F (2,65) =3.8 P<0.05	F (2,65) =0.12 NS
**Treatm x Geno x Site**	F (6,195) =1.48 NS	F (6,195) =0.03 NS	F (6,195) =0.48 NS	F (6,195) =0.23 NS	F (6,195) =0.15 NS	F (6,195) =0.34 NS
**Treatm x Method**	F (3,195) =13.77 P<0.001	F (3,195) =0.39NS	F (3,195) =1.0 NS	F (3,195) =2.73 P<0.05	F (3,195) =17.56 P<0.001	F (3,195) =0.54 NS
**Treatm x Method x Site**	F (6,195) =3.09 P<0.01	F (6,195) =0.4 NS	F (6,195) =4.8 P<0.001	F (6,195) =0.77 NS	F (6,195) =3.75 P<0.005	F (6,195) =0.29 NS
**Treatm x Method x Geno**	F (3,195) =2.72 P<0.05	F (3,195) =1.3 NS	F (3,195) =10.6 P<0.001	F (3,195) =2.3 NS	F (3,195) =10.33 P<0.001	F (3,195) =0.20 NS
**Treatm x Method x Geno x Site**	F (6,195) =1.11 NS	F (6,195) =0.36 NS	F (6,195) =1.56 NS	F (6,195) =0.51 NS	F (6,195) =3.94 P<0.005	F (6,195) =0.11 NS
